# Supplementary material for: Light promoted brown staining of protoplasm by Ag+ is ideal to test wheat pollen viability rapidly
Source: PLoS One. 2020 Dec 16;15(12):e0243856. doi: 10.1371/journal.pone.0243856 (PMC7743923; doi:10.1371/journal.pone.0243856)
Supplement: Attachment — Submitted filename: PONE-D-20-16107_reviewer.pdf [file pone.0243856.s001.pdf]

PLOS ONE

Potential to generate Ag-nanoparticles can be used as an ideal tool to determine pollen viability

--Manuscript Draft--

**Manuscript Number:**	PONE-D-20-16107
**Article Type:**	Research Article
**Full Title:**	Potential to generate Ag-nanoparticles can be used as an ideal tool to determine pollen viability
**Short Title:**	Viable pollen generate Ag-nanoparticles
**Corresponding Author:**	Peddisetty Pardha-Saradhi, Ph.D.<br>University of Delhi<br>Delhi, Delhi INDIA
**Keywords:**	Ag-nanoparticles; Biotransformation; Photoreduction; Pollen viability; Wheat.
**Abstract:**	Pollen viability is crucial for plant breeding programs as it ensures the delivery of functional male gametes to the embryo sac. Present investigations were initiated with an aim to furnish an ideal protocol for unambiguously testing pollen viability. The unique potential of protoplasm of live cells to turn brown due to synthesis of silver nanoparticles (AgNPs) on incubation with medium containing Ag + , in presence of light was exploited for testing pollen viability. Ag + viability test medium incubated with wheat pollen turned brown within 2 min in light, but not in dark. Light microscopic studies showed the presence of uniformly brown protoplasm in cells of viable pollen incubated with Ag + medium in presence of light. Subsequent to rupturing pollen, the brown solution showed AgNPs specific surface plasmon resonance band in its absorption spectrum. Transmission electron microscopic investigations showed the presence of 5-35 nm NPs composed of Ag. Powder X-ray diffraction analysis revealed that AgNPs were biphasic composed of Ag 0 and Ag 2 O. These findings prompted us to evaluate if pollen viability in other angiosperms can be tested with Ag + viability medium. Amazingly, viable pollen from all plant species belonging to 15 distinct families turned brown on incubation with medium with Ag + in presence of light within 2 to 5 min . Conversely, aged non-viable pollen and heat killed non-viable pollen did not turn brown on incubation with Ag + medium in light. We believe that viable pollen possesses unique reducing potential to biotransform Ag + to AgNPs in light. Our findings furnish a novel simplest, rapid, economically viable and green method for testing pollen viability.
**Order of Authors:**	Abhishek Biswas
	Subramaniyan Divya
	Peddisetty Sharmila
	Peddisetty Pardha-Saradhi, Ph.D.
**Additional Information:**	
**Question**	**Response**
**Financial Disclosure**<br><br>Enter a financial disclosure statement that describes the sources of funding for the work included in this submission. Review the submission guidelines for detailed requirements. View published research articles from *PLOS ONE* for specific examples.<br><br>This statement is required for submission	No<br>The funders had no role in study design, data collection and analysis, decision to publish, or preparation of the manuscript.

Powered by Editorial Manager® and ProduXion Manager® from Aries Systems Corporation

and **will appear in the published article** if the submission is accepted. Please make sure it is accurate.<br><br>**Unfunded studies**<br>Enter: *The author(s) received no specific funding for this work.*<br><br>**Funded studies**<br>Enter a statement with the following details:<br>• Initials of the authors who received each award<br>• Grant numbers awarded to each author<br>• The full name of each funder<br>• URL of each funder website<br>• Did the sponsors or funders play any role in the study design, data collection and analysis, decision to publish, or preparation of the manuscript?<br>• **NO** - Include this sentence at the end of your statement: *The funders had no role in study design, data collection and analysis, decision to publish, or preparation of the manuscript.*<br>• **YES** - Specify the role(s) played.<br><br>* typeset	
**Competing Interests**<br><br>Use the instructions below to enter a competing interest statement for this submission. On behalf of all authors, disclose any competing interests that could be perceived to bias this work—acknowledging all financial support and any other relevant financial or non-financial competing interests.<br><br>This statement **will appear in the published article** if the submission is accepted. Please make sure it is accurate. View published research articles from *PLOS ONE* for specific examples.	No<br>The authors have declared that no competing interests exist.

**NO authors have competing interests**<br><br>Enter: *The authors have declared that no competing interests exist.*<br><br>**Authors with competing interests**<br><br>Enter competing interest details beginning with this statement:<br><br>*I have read the journal's policy and the authors of this manuscript have the following competing interests: [insert competing interests here]*<br><br>* typeset	
**Ethics Statement**<br><br>Enter an ethics statement for this submission. This statement is required if the study involved:<br><br>• Human participants<br>• Human specimens or tissue<br>• Vertebrate animals or cephalopods<br>• Vertebrate embryos or tissues<br>• Field research<br><br>Write "N/A" if the submission does not require an ethics statement.<br><br>General guidance is provided below. Consult the submission guidelines for detailed instructions. **Make sure that all information entered here is included in the Methods section of the manuscript.**	N/A

Powered by Editorial Manager® and ProduXion Manager® from Aries Systems Corporation

**Format for specific study types**<br><br>**Human Subject Research (involving human participants and/or tissue)**<br>• Give the name of the institutional review board or ethics committee that approved the study<br>• Include the approval number and/or a statement indicating approval of this research<br>• Indicate the form of consent obtained (written/oral) or the reason that consent was not obtained (e.g. the data were analyzed anonymously)<br><br>**Animal Research (involving vertebrate animals, embryos or tissues)**<br>• Provide the name of the Institutional Animal Care and Use Committee (IACUC) or other relevant ethics board that reviewed the study protocol, and indicate whether they approved this research or granted a formal waiver of ethical approval<br>• Include an approval number if one was obtained<br>• If the study involved *non-human primates*, add *additional details* about animal welfare and steps taken to ameliorate suffering<br>• If anesthesia, euthanasia, or any kind of animal sacrifice is part of the study, include briefly which substances and/or methods were applied<br><br>**Field Research**<br><br>Include the following details if this study involves the collection of plant, animal, or other materials from a natural setting:<br>• Field permit number<br>• Name of the institution or relevant body that granted permission	
**Data Availability**<br><br>Authors are required to make all data underlying the findings described fully available, without restriction, and from the time of publication. PLOS allows rare exceptions to address legal and ethical concerns. See the PLOS Data Policy and FAQ for detailed information.	Yes - all data are fully available without restriction

A Data Availability Statement describing where the data can be found is required at submission. Your answers to this question constitute the Data Availability Statement and **will be published in the article**, if accepted.<br><br>**Important:** Stating 'data available on request from the author' is not sufficient. If your data are only available upon request, select 'No' for the first question and explain your exceptional situation in the text box.<br><br>Do the authors confirm that all data underlying the findings described in their manuscript are fully available without restriction?	
**Describe where the data may be found in full sentences. If you are copying our sample text, replace any instances of XXX with the appropriate details.**<br><br>• If the data are **held or will be held in a public repository**, include URLs, accession numbers or DOIs. If this information will only be available after acceptance, indicate this by ticking the box below. For example: *All XXX files are available from the XXX database (accession number(s) XXX, XXX.).*<br>• If the data are all contained **within the manuscript and/or Supporting Information files**, enter the following: *All relevant data are within the manuscript and its Supporting Information files.*<br>• If neither of these applies but you are able to provide **details of access elsewhere**, with or without limitations, please do so. For example:<br><br>*Data cannot be shared publicly because of [XXX]. Data are available from the XXX Institutional Data Access / Ethics Committee (contact via XXX) for researchers who meet the criteria for access to confidential data.*<br><br>*The data underlying the results presented in the study are available from (include the name of the third party*	All relevant data are within the manuscript

and contact information or URL).<br>• This text is appropriate if the data are owned by a third party and authors do not have permission to share the data.<br><br>* typeset	
Additional data availability information:	

Powered by Editorial Manager® and ProduXion Manager® from Aries Systems Corporation

Manuscript

Click here to access/download;Manuscript;Biswas et al 2020 - Full Manuscript.pdf

Potential to generate Ag-nanoparticles can be used as an ideal tool to determine pollen viability

Abhishek Biswas[#], Subramaniyan Divya[#], P. Sharmila and P. Pardha-Saradhi*

Department of Environmental Studies, University of Delhi, Delhi – 110007, India

*** Corresponding Author**

Email: ppsaradhi@gmail.com

[#]Both Biswas and Divya contributed equally for this manuscript

ABSTRACT

Pollen viability is crucial for plant breeding programs as it ensures the delivery of functional male gametes to the embryo sac. Present investigations were initiated with an aim to furnish an ideal protocol for unambiguously testing pollen viability. The unique potential of protoplasm of live cells to turn brown due to synthesis of silver nanoparticles (AgNPs) on incubation with medium containing Ag^+, in presence of light was exploited for testing pollen viability. Ag^+ viability test medium incubated with wheat pollen turned brown within 2 min in light, but not in dark. Light microscopic studies showed the presence of uniformly brown protoplasm in cells of viable pollen incubated with Ag^+ medium in presence of light. Subsequent to rupturing pollen, the brown solution showed AgNPs specific surface plasmon resonance band in its absorption spectrum. Transmission electron microscopic investigations showed the presence of 5-35 nm NPs composed of Ag. Powder X-ray diffraction analysis revealed that AgNPs were biphasic composed of Ag^0 and Ag_2O. These findings prompted us to evaluate if pollen viability in other angiosperms can be tested with Ag^+ viability medium. Amazingly, viable pollen from all plant species belonging to 15 distinct families turned brown on incubation with medium with Ag^+ in presence of light within 2 to 5 min. Conversely, aged non-viable pollen and heat killed non-viable pollen did not turn brown on incubation with Ag^+ medium in light. We believe that viable pollen possesses unique reducing potential to biotransform Ag^+ to AgNPs in light. Our findings furnish a novel simplest, rapid, economically viable and green method for testing pollen viability.

KEYWORDS: Ag-nanoparticles; Biotransformation; Photoreduction; Pollen viability;

INTRODUCTION

Pollen, the male gametophytes of phanerogams (seed yielding plants), constitute one of the two key parts of sexual reproduction. Pollen grains, which vary significantly in shape and size depending on the plant species, are pollinated/dispersed to the destination (i.e. female organs that bear female gametophyte) through specific channel(s) such as wind, insects and birds. Pollen viability signifies the ability of pollen to bring about fertilization by providing functional male gametes. Pollen viability is critical not only for natural cross/self-fertilization, but also for specific breeding programs that requires precise self or cross pollination. Besides playing a crucial role in general plant breeding/hybridization involving transfer of unique genes that impart abiotic/biotic stress tolerance/resistance or enhance nutritional quality from elite genotypes, pollen/microspore viability plays pivotal role in (i) hybrid seed production; (ii) haploid/double-haploid technology; (iii) genetic engineering; (iv) determining transgene contamination; and (v) species dispersal, fitness and survival/continuation of plant species [1-6]. Viability of pollen, which is considered to be an index of its quality and vigor, after their release from the anther, varies greatly from species to species. Pollen viability ranges from few minutes to few years depending on the taxonomic status of the plant and/or the abiotic environmental factors [6,7]. Special storage conditions are needed to maintain the viability and fertilizing ability of the pollen for a long duration [6].

Taking into account the immense role of pollen in agriculture and forestry, it is important to monitor their viability from time to time. Although a number of protocols have been developed for evaluating viability of pollen grains, each protocol has limitation [4-6]. Even though, pollen germination test is considered to be ideal for evaluating viability, researchers failed to germinate viable pollen of several plant species due to the requirement of some specific conditions or immaturity. As a result researchers identified alternate protocols that test/detect (i) the dehydrogenase (an enzyme that is omnipresent in all living cells) activity using a 2,3,5-triphenyl tetrazolium chloride or 2,5-diphenyl monotetrazolium bromide, where viable pollen turn deep pink; (ii) callose in pollen walls and tubes with aniline blue (aniline blue in lactophenol); (iii) starch using Lugol solution (which contain iodine in KI), where viable pollen turns black; (iv) chromatin using Acetocarmine; (v) cytoplasm and cell wall using the Alexander stain, where viable pollen's cytoplasm turns red, while the cell wall turns green; (vi) the esterase activity by means of fluorochromatic reaction, where fluorescence emitted within viable pollen by fluorescein released by esterase from fluorescein diacetate can be viewed under a fluorescence microscope; and (vii) the peroxidase activity using Phenylenediamine, which is oxidized in presence of H_2O_2 to produce diaminophenazine that turns viable pollen

black [1-6,8]. Due to difficulty and lack of consistency in these methods, Heidmann et al. [6] used reliable impedance flow cytometry (IFC) for testing pollen viability. However, IFC test essentially requires (i) filtration of pollen suspended in a specific IFC measurement buffer through specific filters; (ii) loading of filtered pollen in IFC buffer onto pollen size specific channel chips; (iii) insertion of chips into IFC; (iv) measurement of changes of electrical impedance as pollen pass through dual interrogation points in response to an alternating current at four user-defined frequencies in the mid and high frequency bands, using special software like AmphaSoft v1.2. Although, this method is reliable, its use would be limited due to its complexity, expensiveness and most importantly requirement of special equipment.

In light of limitations in prevailing pollen viability protocols, it is important to develop novel and reliable protocol(s) for testing pollen viability. Previously we reported that living cells and chloroplasts have potential to generate silver nanoparticles as they possess strong reducing strength [9-11]. These findings from our team prompted us to evaluate if pollen viability can be tested based on their potential to generate AgNPs from Ag^{+}. It is known that pollen possess (i) carbohydrates (in particular starch and sugars), lipids and proteins; and (ii) enzymes such as dehydrogenases in their protoplasm [3-6,12,13]. These biomolecules including dehydrogenases can promote reduction Ag^{+} to generate AgNPs [9,14-16] and hence can turn protoplasm brown. Generation of AgNPs from Ag^{+} basically relies on reduction by reducing agents that have higher negative redox potential compared to Ag^{+} [9,15]. In this communication we are reporting for the first time that (i) the protoplasm of pollen cells turn brown due to its capacity to promote reduction of Ag^{+} to generate biphasic AgNPs; and (ii) pollen viability can be tested by evaluating their potential to generate AgNPs.

MATERIALS AND METHODS

Collection of pollen grains: Pollen grains were collected on butter paper from potted plants in our laboratory complex, Department of Environmental Studies, University of Delhi as well as from the Gardens of University of Delhi, generally in the morning hours between 8 to 10 AM. Pollen collected from bread wheat (*Triticum aestivum* cv. 1544, Poaceae), was used for standardizing the composition of testing medium and also for detailed analysis. Pollen samples were also collected from pigeon pea (*Cajanus cajan* cv. 951, Fabaceae), Indian mustard (*Brassica juncea* cv. Varuna, Brassicaceae), onion (*Allium cepa*, Amaryllidaceae), fennel (*Foeniculum vulgare*, Apiaceae), sunn hemp (*Crotalaria juncea*, Fabaceae), flaming glorybower (*Clerodendrum splendens*, Lamiaceae), garden nasturtium (*Tropaeolum majus*, Tropaeolaceae), black nightshade (*Solanum nigrum*, Solanaceae), common hollyhock (*Alcea rosea*,, Malvaceae), garden phlox (*Phlox paniculate*, Polemoniaceae), garden petunia (*Petunia*

axillaris, Solanaceae), rainbow pink (*Dianthus chinensis*, Caryophyllaceae), dahlia (*Dahlia pinnata*, Asteraceae), Mexican marigold (*Tagetes erecta*, Asteraceae), African daisy (*Dimorphotheca pluvialis*, Asteracea), poppy (*Papaver rhoeas*, Papaveracea), snapdragon (*Antirrhinum majus*, Plantaginaceae), China rose (*Hibiscus rosa-sinensis*, Malvaceae) and metal leaf begonia (*Begonia metallica*, Begoniaceae).

Standardization of pollen viability medium: For testing potential of pollen to reduce Ag^+ to generate AgNPs, $AgNO_3$ and Ag_2SO_4 (Merck Specialties Pvt. Ltd., India) were used. For maintaining desired osmolarity to prevent bursting of pollen different osmotic agents namely KNO_3, KCl, sucrose and glycerol (Merck Specialties Pvt.Ltd., India) were used. Preliminary, but authentic studies revealed that (i) both $AgNO_3$ and Ag_2SO_4 promoted generation of AgNPs, but former was superior in rapidly turning test solution brown due to generation of AgNPs; (ii) 0.5 mM $AgNO_3$ was superior amongst different concentrations namely 0.25, 0.5 and 1 mM tested; and (iii) 300 mM KCl gave consistent results, amongst different osmotic agents tested. However, (i) on mixing $AgNO_3$ with KCl, an opaque white colloidal solution was produced as a result of formation of AgCl within a short duration; (ii) with duration of storage, efficacy of $AgNO_3$+KCl mixture to turn pollen evenly and intensely brown decreased. Best, authentic and most reliable results were obtained when pollen suspended in 100 µl of 600 mM KCl were treated with 100 µl of 1 mM $AgNO_3$ (resultant mixture of KCl and $AgNO_3$ is hereafter referred as Ag^+ viability test medium), so that the final and effective concentration of $AgNO_3$ and KCl remain 0.5 and 300 mM, respectively. Therefore, it is important to disclose specifically that 1 mM $AgNO_3$ and 600 mM KCl solutions must be stored separately and former solution be added soon after pollen were incubated in later solution.

Evaluating novel Ag^+ medium for testing pollen viability: Within 10-20 min after collection, few pollen grains were incubated in Ag^+ viability medium (as specified above) and exposed to solar radiation or high intensity visible light with a photon flux density (PFD) of ~600 µmol $m^{-2}s^{-1}$, for 1 to 5 min under ambient conditions. In order to establish the necessity of light for photoreduction of Ag^+ by protoplasm of pollen cells to brown AgNPs, an independent set of pollen grains in viability medium were incubated in dark. Subsequently, the pollen grains were observed under Olympus CX40RF200 trinocular microscope (Olmypus Optical Co. Ltd., Japan). Images of pollen of all plant species exposed to different treatments were grasped using Olympus PEN E-PL1 camera (Olympus Imaging America Inc., USA) attached to the microscope at different magnifications.

Characterization of AgNPs: Subsequent to incubation, the medium with pollen was diluted by adding equal proportion of distilled water to promote bursting of pollen. Following sonication,

the absorption spectra of resultant solutions were recorded using UV-Vis spectrophotometer (Analytikjena Specord 200, Jena, Germany) at a resolution of 10 nm and a scan rate of 10 nm/sec. For TEM investigations, 10 µl of the sonicated solution was drop coated onto a 200 mesh copper TEM grid with an ultrathin continuous carbon film and allowed to dry in a desiccator. The grids were viewed in the transmission electron microscope (Technai G2 T30 U-TWIN, Lonate Pozzolo, Italy) at a voltage of 300 kV. The hardware associated with this instrument allowed energy dispersive X-ray (EDX) analysis to measure the elemental composition. For Powder X-ray diffraction (PXRD) studies the brown sonicated colloidal solution was centrifuged at 10,000 x*g* and the resultant pellet was suspended in minimum quantity of double distilled water. Subsequently this suspension was drop coated on the silica surface and after drying in a desiccator under ambient conditions, the PXRD pattern was collected using Rigaku Rotaflex RAD-B (Rigaku Analytical Devises, Inc., USA) with copper target CuK(α)1 radiation at the rate of 0.020 step in 1.2 s in 2 theta (θ) range of 10–70º, with a tube voltage of 40 kV and a current of 60 mA.

All experiments, with the exception of TEM and PXRD analysis, were carried out independently at least six times.

RESULTS AND DISCUSSION

Wheat pollen turned $AgNO_3$ and Ag_2SO_4 solutions brown within few seconds on incubation in presence of solar radiation or high intensity light (having a photon flux density of ~600 µmol $m^{-2}s^{-1}$). Microscopic observations revealed the presence of only burst pollen suspended in brown colloidal matter. It is well known that pollen burst, when placed in water or any hypotonic solution [17] as net movement of water into pollen (i.e. absorption of water by pollen) occurs through germ pore(s)/aperture(s), which possess thin wall made of intine with very thin or no exine. Instantaneous diffusion of stained/coloured protoplasm of pollen cells into surrounding hypotonic viability test medium has been recorded by earlier researchers also [17]. To overcome this problem, pollen viability test media are enriched with various components such as sugars like sucrose and/or salts like KNO_3, KCl etc. to enhance their osmotic strength and maintain desired osmotic balance [17]. During present investigations, amongst different components viz. sucrose, glycerol, KNO_3 and KCl tested, 600 mM KCl and 1 mM Ag^+ in the ratio of 1:1, was found to be most suitable for staining wheat pollen homogenously brown.

Ag^+ viability medium with wheat pollen grains turned brown within 2 min on incubation under ambient conditions in presence of solar radiation or high intensity visible light, but not in dark (Fig 1A). As evident from Fig 1B, protoplasm of pollen turned evenly brown on incubation in

medium containing Ag^+, only in presence of solar radiation or high intensity visible light. It is well established that solution containing Ag^+ turns brown due to generation of AgNPs [9-11,14-16]. Subsequent to incubation of pollen in osmotically ideal (that maintain integrity of pollen) media containing 0 and 0.5 mM Ag^+ in presence and absence of light, the media were diluted with equal proportion of distilled water to create hypotonic conditions (i.e. osmotic imbalance) that promote release pollen contents due to bursting, in order to make the media relatively homogenously colloidal pale yellow (control) or brown (Ag^+ treated). Absorption spectra of sonicated resultant colloidal suspensions are depicted in Fig 1C. Brown colloidal suspension obtained from Ag^+ viability medium incubated with wheat pollen in light showed a prominent peak around ~420 nm (Fig 1C). It is well known that this peak around ~420 nm arises due to surface plasmon resonance specific to AgNPs [9-11], which ascertained the potential of wheat pollen to generate AgNPs rapidly on incubation in Ag^+ viability medium in presence of high intensity light or solar radiation.

TEM investigations confirmed the presence of distinct 5 to 35 nm nanoparticles comprised of Ag, in brown solutions that resulted due to incubation of pollen in Ag^+ viability medium in presence of light (Fig 1D). The PXRD pattern of brown colloidal matter showed Bragg reflections at (a) (111), (200) and (311) revealing the face centered cubic structure of Ag^0NPs; and (b) (111)*, (211)*, (220)* and (221)* corresponded to cubic Ag_2O (Fig 1E). Thus, PXRD studies clearly revealed that AgNPs were biphasic, composed of both Ag^0 and Ag_2O. It is well-known that Ag^0 and Ag^0NPs are prone to oxidation, which results in the generation of Ag_2ONPs [9-11].

Previously, we reported potential of (i) live leaf cells; and (ii) chloroplasts to reduce Ag^+ to generate AgNPs in presence of solar radiation or high intensity light (~600 µmol m^{-2} s^{-1} PFD) under ambient conditions [10,11]. Heat killed wheat pollen incubated in Ag^+ viability medium in presence of solar radiation or high intensity light failed to turn brown (fig. 1D), revealing the necessity of live components within pollen for rapid generation of AgNPs.

In order to check, if Ag^+ viability medium can be used for testing pollen viability in all plant species, we extended our studies to 20 plant species (namely pigeon pea, Indian mustard, fennel, sunn hemp, flaming glorybower, garden nasturtium, black nightshade, common hollyhock, garden phlox, garden petunia, rainbow pink, dahlia, Mexican marigold, African daisy, poppy, snapdragon, China rose, metal leaf begnonia and onion including wheat) from 15 diverse families of angiospermsm which included Fabaceae, Brassicaceae, Apiaceae, Lamiaceae, Tropaeolaceae, Solanaceae, Malvaceae, Polemoniaceae, Caryophyllaceae, Asteraceae, Papaveraceae, Plantaginaceae, Begoniaceae, Amaryllidaceae and Poaceae.

Interestingly, protoplasm of viable pollen of all the 20 plant species from distinct families turned more or less evenly brown, when incubated in Ag^+ viability medium in presence of solar radiation or high intensity light within 2-5 min (Fig 1B & Fig 2A-2K). However, intensity and shade of brown colour varied significantly amongst plant species. Heat killed and non-viable pollen grains of these plant species failed to turn brown on incubation in Ag^+ viability medium in presence of solar radiation or high intensity light (Fig 1F & Fig 2L-2O). These findings, further establish that active/live protoplasm is necessary to turn pollen brown due to potential to generate AgNPs.

Mechanism of brown staining of viable pollen through generation of AgNPs: Pollen, the male gametophyte, represents one of the most vital stages in life cycle of phanerogams exhibit vigorous metabolism. Accordingly, they are enriched with a range of biomolecules, which include starch, sugars, lipids, proteins and citrate [3-6,12,13,17,19] for generating desired energy, redox power and range of carbon skeletons. Several such biomolecules promote photoreduction and biotransformation of Ag^+ to AgNPs [10,18,19]. Guo and co-workers [18] recorded sunlight promoted biotransformation of Ag^+ into AgNPs by biomolecules from root exudates embroiling (i) generation of AgCl microcubes; (ii) stabilization of these AgCl microcubes by biomolecules; and (iii) photoreduction of Ag^+ in AgCl miccrocubes to Ag^0NPs by biomolecules. However, Ag^0 as well as Ag^0NPs are prone to oxidation under aerobic conditions [9-11] and accordingly, as evident from PXRD analysis (Fig. 1E) AgNPs generated by pollen through photoreduction are biphasic made of Ag^0 and Ag_2O. We have earlier reported that dehydrogenases promote reduction Ag^+ to generate AgNPs [9] and pollen possess dehydrogenases [2-6,17]. Therefore, we strongly believe that viable pollen, which invariably exhibit active metabolism, generate biphasic AgNPs through photoreduction using inbuilt biomolecules and dehydrogenases. These biphasic AgNPs turn the protoplasm of viable pollen cells brown and hence Ag^+ viability medium can be used authentically for universally testing pollen viability.

In summary, we developed an authentic Ag^+ pollen viability test, which involves (i) simultaneous addition of 600 mM KCl and 1 mM $AgNO_3$ or Ag_2SO_4 to pollen, followed by incubation in presence of solar radiation or high intensity light (~600 µmol photon flux density $m^{-2}s^{-1}$) for 2 min under ambient conditions; and (ii) light microscopic recording of brown staining of viable pollen. This Ag^+ pollen viability test could be successfully extended to a range of angiosperms belonging to 15 diverse taxonomic families. As this Ag^+ pollen viability test failed to stain heat killed and non-viable pollen brown, we strongly believe that active metabolism in viable pollen promote photoreduction and biotransformation of Ag^+ to AgNPs.

To the best of our knowledge, the novel Ag^+ pollen viability test developed by us is the simplest, rapid, economically viable and green authentic method for testing pollen viability.

Authors Contribution

Conceived and designed the experiments: PPS. Performed the experiments: PPS AB SD PS. Analyzed the data: PPS AB SD PS. Contributed reagents/materials/analysis tools: PPS PS. Wrote the paper: PPS ~~DS~~ AB PS.

Competing Interests: The authors have declared that no competing interests exist.

References

1. Stone JL, Thomson JD, Dent-Acosta SJ. Assessment of pollen viability in hand-pollination experiments: a review. American Journal of Botany, 1995; 82:1186-1197 doi: 10.1002/j.1537-2197.1995.tb11591.x
2. Dafni A ~~&~~ Firmage D. Pollen viability and longevity: practical, ecological and evolutionary implications. Plant Systematics and Evolution, 2000; 222, 113-132 doi: 10.1007/BF00984098
3. Firon N, Nepi M, Pacini E. Water status and associated processes mark critical stages in pollen development and functioning. Annals of Botany, 2012; 109, 1201–1214. doi: 10.1093/aob/mcs070
4. Heidmann I, Schade-Kampmann G, Lambalk J, Ottiger M, Di Berardino M. Impedance flow cytometry: a novel technique in pollen analysis. PLoS One, 2016; 11: 0165531 doi: 10.1371/journal.pone.0165531 pmid:27832091
5. Impe D, Reitz J, Köpnick C, Rolletschek H, Börner A, Senula A, Nagel M. Assessment of pollen viability for wheat. Frontiers in Plant Science, 2020; 10: 1588 doi: 10.3389/fpls.2019.01588 pmid:32038666
6. Pacini E ~~and~~ Dolferus R. Pollen developmental arrest: maintaining pollen fertility in a world with a changing climate. Frontiers in Plant Science, 2019; 10:679. doi: 10.3389/fpls.2019.00679
7. Mesihovic A, Iannacone R, Firon N, Fragkostefanakis S. Heat stress regimes for the investigation of pollen thermotolerance in crop plants. Plant Reproduction, 2016; 29, 93–105. doi: 10.1007/s00497-016-0281-y
8. Rodriguez-Riano T, ~~&~~ Dafni A. A new procedure to asses pollen viability. Sexual Plant Reproduction, 2000; 12(4), 241-244 doi: 10.1007/s004970050008

9. Pardha-Saradhi P, Yamal G, Peddisetty T, Sharmila P, Nagar S, Singh J, Nagarajan R, Rao K.S. Reducing strength prevailing at root surface of plants promotes reduction of Ag^+ and generation of Ag^0/Ag_2O nanoparticles exogenously in aqueous phase. PLoS ONE, 2014; 9: 106715 doi: 10.1371/journal.pone.0106715 pmid:25184239
10. Shabnam N, Sharmila P, Kim H, Pardha-Saradhi P. Light Mediated Generation of Silver Nanoparticles by Spinach Thylakoids/ Chloroplasts. PLoS ONE, 2016; 11: 0167937 doi: 10.1371/journal.pone.0167937 pmid:27936248
11. Shabnam N, Sharmila P, Govindjee, Kim H, ~~&~~ Pardha-Saradhi P. Differential response of floating and submerged leaves of longleaf pondweed to silver ions. Frontiers in Plant Science, 2017; 8, 1052. doi: 10.3389/fpls.2017.01052
12. Pardha-Saradhi P, & Ram HYM. Some aspects of floral biology of Cassia fistula Linn.(The Indian laburnum)-Part 1. Current Science, 1981; 50: 802-805.
13. Speranza A, Calzoni GL, Pacini EL. Occurrence of mono- or disaccharides and polysaccharides reserve in mature pollen grain. Sexual Plant Reproduction 1997; 10: 110-115 doi: 10.1007/s004970050076
14. Beattie IR and Haverkamp RG. Silver and gold nanoparticles in plants: sites for the reduction to metal. Metallomics, 2011; 3: 628-632 doi: 10.1039/c1mt00044f. pmid:21611658
15. Yamal G, Sharmila P, Rao KS, Pardha-Saradhi P. Inbuilt potential of YEM medium and its constituents to generate Ag/Ag_2O nanoparticles. PLoS ONE, 2013; 8: 61750 doi: 10.1371/journal.pone.0061750 pmid:23626722
16. Mohan S, Oluwafemi OS, George SC, Jayachandran VP, Lewu FB, Songca SP, Kalarikkal N, Thomas S. Completely green synthesis of dextrose reduced silver nanoparticles, its antimicrobial and sensing properties. Carbohydrate Polymers, 2014; 106: 469-474 pmid:24721103
17. Heslop-Harrison J, Heslop-Harrison Y & Shivanna K R. The evaluation of pollen quality, and a further appraisal of the fluorochromatic (FCR) test procedure. Theoretical and Applied Genetics, 1984; 67: 367–375. doi:10.1007/bf00272876
18. Guo H, Ma C, Thistle L, Huynh M, Yu C, Clasby D, Chefetz B, Polubesova T, White JC, He L, Xing B. Transformation of Ag ions to Ag nanoparticles-loaded AgCl microcubes in the plant root zone. Environmental Science: Nano, 2019; 6: 1099-1110. doi:10.1039/c9en00088g

19. Maillard M, Huang P, Brus L. Silver Nanodisk Growth by Surface Plasmon Enhanced Photoreduction of Adsorbed [Ag^+]. Nano Letters, 2003; 3: 1611–1615. doi:10.1021/nl034666d

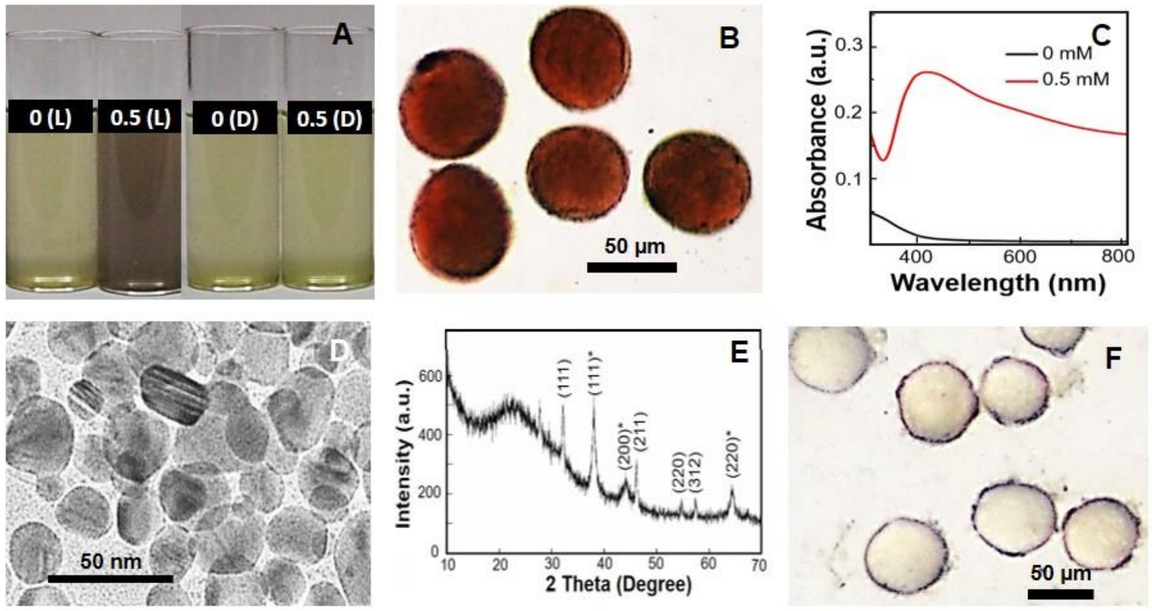

Fig 1. Potential of wheat pollen to generate AgNPs: (A)Variation in color of viability medium with 0 and 0.5 mM Ag^+ incubated with wheat pollen in presence of solar radiation (l) and dark (d) for 5 min; (B) Light microscopic image of wheat pollen showing brown stain; (C) Absorption spectra of sonicated media with wheat pollen incubated with 0 and 0.5 mM Ag^+ [revealing presence of AgNPs specific Plasmon resonance peak around 420 nm] in presence of solar radiation; (D) Transmission electron micrograph showing the presence of distinct AgNPs in the sonicated pollen after incubation with Ag^+ viability medium in presence of light; (E) PXRD pattern of AgNPs formed by the wheat pollen incubated with Ag^+ viability medium in presence of light. Bragg reflections confirmed that these AgNPs are biphasic composed of Ag^0 '()' and Ag_2O '()*'; (F) Heat killed wheat pollen incubated with Ag^+ viability medium in presence of light, which reveals the necessity of live/active pollen for brown staining by generating AgNPs.

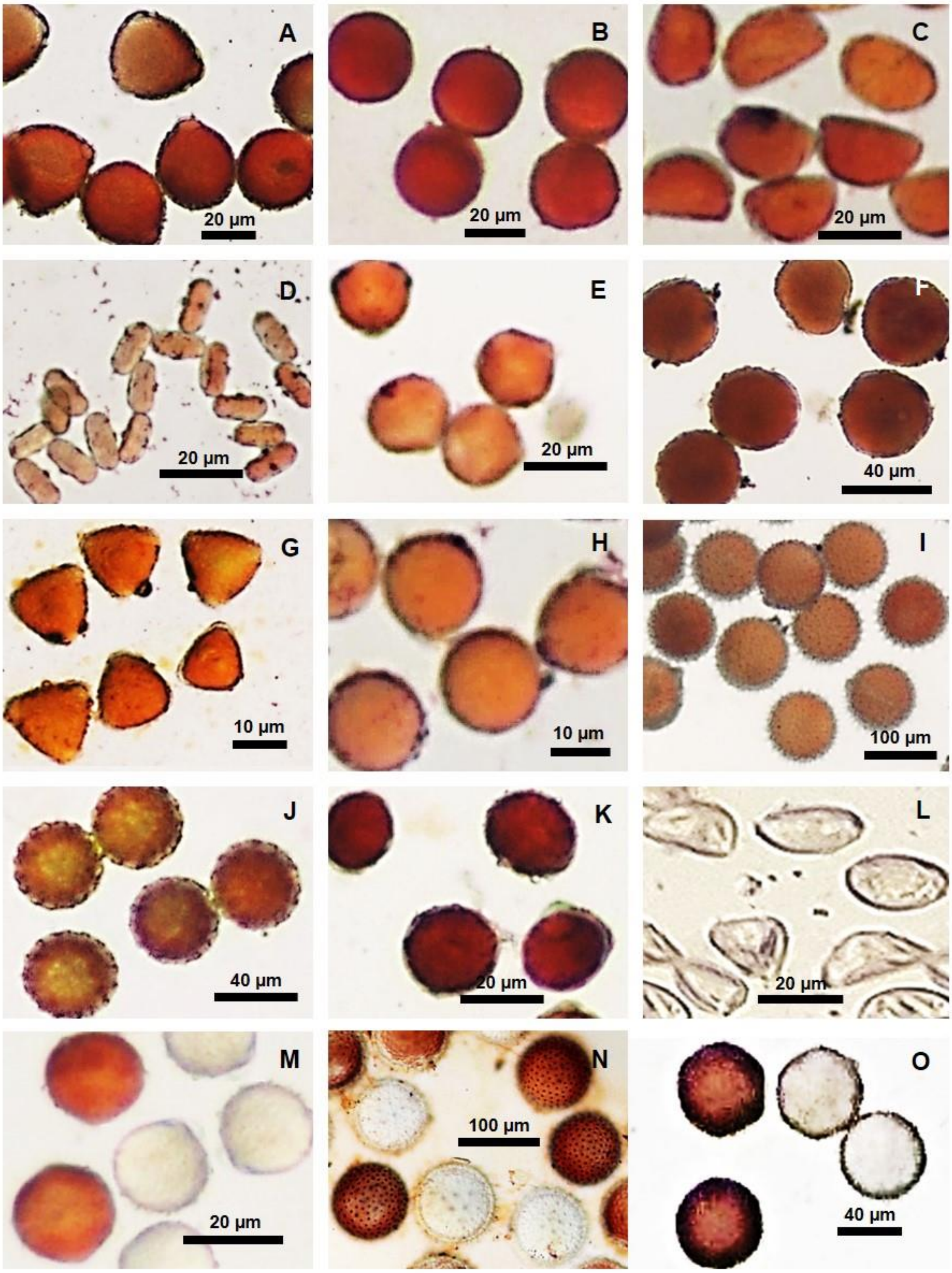

Fig 2. Potential of Ag^+ viability medium to stain viable pollen brown: Light micrograph of pollen of (A) pigeon pea; (B) Indian mustard; (C) onion; (D) fennel; (E) ~~C~~sunn hemp; (F) flaming glorybower; (G) garden nasturtium; (H) black nightshade; (I) common hollyhock; (J) common hollyhock; (K) garden petunia; (L) onion; (M) sunn hemp; (N) dahlia; and (O) rainbow pink incubated in Ag^+ viability medium (composed of freshly added 600 mM KCl to

pollen followed by simultaneous addition of 1 mM $AgNO_3$ in equal proportion) in presence of high intensity visible light (~600 µmol photon flux density $m^{-2}s^{-1}$) for 2 min. (A) to (K) and (M) to (O) show brown staining of viable pollen of various Angiosperms, due to generation of AgNPs through photoreduction of Ag^+ by inbuilt pollen constituents. Similar results were also obtained when incubated in presence of sunlight. Heat killed pollen of onion (L) failed to turn brown as they lacked potential to generate AgNPs under similar conditions. Similarly, non-viable pollen of sunn hemp (M), dahlia (N) and rainbow pink (O) failed to stain brown as they also lacked potential to generate AgNPs on incubation in Ag^+ viability medium in presence of light. These findings clearly reveal the potential of viable pollen to turn brown due to active metabolism that promote photoreduction of Ag^+ to generate AgNPs
