# Supplementary material for: Light promoted brown staining of protoplasm by Ag+ is ideal to test wheat pollen viability rapidly
Source: PLoS One. 2020 Dec 16;15(12):e0243856. doi: 10.1371/journal.pone.0243856 (PMC7743923; doi:10.1371/journal.pone.0243856)
Supplement: Attachment — Submitted filename: Response to Reviewers -Appeal - PONE-D-20-16107.pdf [file pone.0243856.s002.pdf]

Appeal

[Authors may submit a formal appeal for rejected submissions. Appeal requests must be made in writing to plosone@plos.org with the word "appeal" in the subject line. Authors must provide detailed reasons for the appeal and point-by-point responses to the reviewers' and/or Academic Editor's comments.]

PONE-D-20-16107

Potential to generate Ag-nanoparticles can be used as an ideal tool to determine pollen viability

PLOS ONE

Academic Editor's Comment: "After careful consideration, we have decided that your manuscript does not meet our criteria for publication and must therefore be rejected."

Response*: According to both the Reviewers 1 and 2, the manuscript was presented in an intelligible fashion and written in standard English. Reviewer 1 further stated that "The manuscript is written well and proper justifications are given to the results obtained."*

Further, all the issues raised by the reviewer are minor in nature and can be rectified with ease. To the best of our knowledge, none of the issues raised by the Reviewers are serious enough for "Rejection".

Unfortunately, both the Academic Editor (Dr. Vijay Kumar) as well as Reviewers failed to understand the basic goal/objective of our manuscript. All of them seem to be experts, and interested and accordingly focused on outdated biogenic synthesis of Ag-nanoparticles (especially with extracts of plant parts including pollen). It is surprising that the Academic Editor, Dr. Vijay Kumar failed to give his own independent comments/views [he used only standard Rejection letter of PLoS One, without furnishing any Specific comments for the Rejection]. As none of the comments of the Reviewers are serious in nature, we believe that the "Rejection" decision taken by the Academic Editor, Dr. Vijay Kumar is 100% biased and it only reflects that he has some personal vengeance against us.

By looking at vagueness of the decision and comments, I initially preferred not to be part of PLoS One community any further. But the co-authors insisted me to Appeal to expose biased attitude of the Academic Editor, Dr. Vijay Kumar, to curb such illogical decisions. However, while respecting the comments [albeit many of them are vague], we annotated the manuscript significantly.

As elaborating on the reasons for using each plant/crop species, would unnecessarily (i) dilute the key objective and finding of our investigations; and (ii) increase the length of the manuscript, we decided to restrict to only wheat [as detailed investigations were carried with this plant species] and also furnish data obtained using Ag^+-viability medium consisting of $AgNO_3$ and KNO_3 [and totally omitted the information related with KCl]. In fact, the entire data furnished in the original manuscript was obtained using Ag^+-viability medium consisting of $AgNO_3$ and KNO_3. Although, we obtained similar results even when we replaced KNO_3 with KCl [as both these salts are used as osmotic agents to prevent pollen bursting]. The same has been stated in our original manuscript. Unfortunately, due to negligence we mistakenly typed KCl in place of KNO_3 while disclosing the composition of Ag^+-viability medium. This mistake was committed as we wanted to disclose interesting observation, wherein we recorded staining of pollen brown by $AgNO_3$ even in presence of KCl, although $AgNO_3$+ K Cl mixture turned colloidal due to formation of AgCl [we indeed specifically pointed out the formation of colloidal medium due to the formation of AgCl in our Original manuscript]. Probably the Academic Editor as well as the Reviewer 2 failed to go through our manuscript judiciously. To avoid any confusion, now we deleted KCl results. Please find below our comments on every point raised by both the Reviewers.

Reviewer #1 (R1)

R1 Comment: The manuscript entitled "Potential to generate Ag-nanoparticles can be used as an ideal tool to determine pollen viability" has been reviewed. "It is very general observation that plant cells and extracts could be used for biogenic synthesis of AgNPs."

"The manuscript is written well and proper justifications are given to the results obtained."

***Response**: This Reviewer specifically stated/agreed that the manuscript is written well and proper justifications are given to the results obtained. We fail to understand how can a manuscript that is written well with proper justification to the findings/results can be blindly rejected by the Academic Editor Dr. Vijay Kumar. We accept that the biogenic synthesis of AgNPs by plant cells and extracts is a very general observation. However, objective of our investigations presented in the manuscript is not the general biogenic synthesis of AgNPs. Our aim was to develop a simple method for testing pollen viability. We used Ag^+ for this endeavor and specifically demonstrated that the pollen viability can be tested using $AgNO_3$. However, to explain the mechanism associated with brown staining, we specified that brown staining of pollen on incubation with $AgNO_3$ was*

due to synthesis of AgNPs [which have been characterized for proving the mechanism associated with staining)].

R1 Comment: There are certain points to be rectified before further processing of the manuscript: "• What was the criterion in selection of plants for pollen viability testing?" "• Why these plants only selected?"

Response*: Wheat is the major cereal crop [this information is now incorporated in the annotated manuscript] and continuous efforts are being made across the globe for its improvement through plant breeding program. Viable pollen is the key for successful plant breeding.*

Similarly, pigeon pea is an important pulse crop, Indian mustard is an important oil crop fennel is a spice crop, hemp is an important fiber crop, onion is an important vegetable crop. Black night shade is an important medicinal plant. Others are important floricultural crops, each of which possess some medicinal utility besides aesthetic value. Most importantly, all of them were easily available for our studies without any barrier. We don't believe in increasing manuscript size by describing purpose of using each of them by briefing on importance of each floricultural plant species. In fact, such efforts will dilute the key objective of any useful investigation. We strongly believe that the manuscript should be crisp and focus on findings related to the objective/hypothesis, rather than unnecessarily increasing the length of the manuscript. Therefore, in annotated manuscript, we restricted ourselves to only wheat as we carried detailed and complete investigations with wheat pollen.

In fact, we submitted our manuscript as a Brief Communication to PLoS Biology and the same has been transferred to PLoS One [as an internal policy, after seeking permission of the Authors].

R1 Comment: "• Part of second paragraph of Material and methods section should be presented in Results and discussion section."

"• Even some parts of R and D section can be part of M and M section too."

Response*: Necessary action taken in Annotated manuscript.*

R1 Comment: "• What was the standard solution used for the spectroscopic analysis of AgNP containing pollens?"

Response*: We used mixture containing 0.5 mM $AgNO_3$ and 300 mM KNO_3, which has been diluted by adding equal volume of distilled water, in a manner similar to the way viability medium incubated with pollen was diluted. The later has been stated clearly in our manuscript.*

R1 Comment: "• Write in details about the data analysis of this study."
Response*: We have already furnished information on data analysis. It is not clear what additional 'details about the data analysis' are required. However, we have further elaborated Methods.*

R1 Comment: "• Ag+ viability medium with wheat pollen grains turned brown within 2 min on incubation under ambient conditions in presence of solar radiation or high intensity visible light, but not in dark (Fig 1A). As evident from Fig 1B, protoplasm of pollen turned evenly brown on incubation in medium containing Ag+, only in presence of solar radiation or high intensity visible light. It is well established that solution containing Ag+ turns brown due to generation of AgNPs"....... The authors must discuss in details about the role of light in formation of AgNPs. Because, they got AgNPs in light conditions only."
Response*: At this stage, it is difficult for us to discuss details about the actual role of light in formation of AgNPs. We stated that high intensity light promoted <u>rapid</u> generation of AgNPs. However, if pollen are incubated under lab conditions for long duration, one can still see alteration in colour of viability medium with pollen, albeit the intensity of brown colour is low. Our objective is to develop a rapid pollen viability test. It is important to again stress that our aim was <u>not</u> to generate AgNPs. Generation of AgNPs by plant extracts has been carried out by innumerable number of investigators (surprisingly, even today such works get published) and in fact presently, generation of AgNPs by plant extracts has become a simple lab experiment for school and college students. At present, such works certainly don't make any sense.*

R1 Comment: "• Sentence should not be started with the abbreviations...."
Response*: Suggested corrections incorporated.*

R1 Comment:"• It is well established fact that living cells and cellular organelles help in biogenesis of AgNPs, therefore, the pollen too helped in this study. Weather such pollens could be used for plant breeding experiments without losing their viability after used in this experiment?"

***Response**: To the best of our knowledge, use of silver salts for testing viability of pollen is novel. Yes, besides few other investigators, our research team has also authentically demonstrated that live cells as well as live chloroplasts possess potential to generate Ag-nanoparticles [some of which have been published in PLoS One itself]. In fact, this is the basic background (hypothesis) based on which we attempted to test if such a potential of live cells to generate Ag-nanoparticles can be exploited for testing pollen viability. It is relevant to cite few classical examples related for testing pollen viability itself. Earlier, few dynamic and pioneering researchers used TTC assay, esterase activity, peroxidase activity, IKI starch staining, acetocarmine test etc., all of which are known to exist in live cells for several decades. But, these dynamic researchers got due credit for using such approaches for testing pollen viability. We also wish to quote a dynamic pollen viability test published in PLOS One itself [Heidmann I, Schade-Kampmann G, Lambalk J, Ottiger M, Di Berardino M. Impedance flow cytometry: a novel technique in pollen analysis. PLoS One 2016;11: 0165531 doi: 10.1371/journal.pone.0165531], wherein Impedance flow cytometry was used for testing pollen viability. In fact, due to importance and relevance, researchers across the globe have been making efforts in developing novel and simple protocols for testing pollen viability. Similarly, recently Impe & co-workers published a paper on assessment of pollen viability in wheat in Frontiers in Plant science [Impe D, Reitz J, C Köpnick, Rolletschek H, Börner A, Senula A, Nagel M. Assessment of pollen viability for wheat. Front Plant Sci. 2020;10: 1588 doi: 10.3389/fpls.2019.01588]. Compared to the protocols used by these investigators as well as others so far, the pollen viability test developed by us is rapid, simplest and cost effective.*

R1 Comment: "• "in presence of solar radiation or high intensity light (Fig 1F & Fig 2L-2O). These findings, further establish that active/live protoplasm is necessary to turn pollen brown due to potential to generate AgNPs".... But there are reports which used plant extracts only to generate AgNPs in the solution within one min.....any clarification in this regard..."

***Response**: It looks that this reviewer failed to understand/grasp the basic hypothesis/objective of our investigations presented in this manuscript. Our aim was certainly not to furnish a protocol for generation of Ag-nanoparticles. Use of plant extracts for generation of Ag-nanoparticles has been known for several decades and presently, it is well known that such findings using blind use of extract of different parts of various plant species has no relevance as plant extracts possess innumerable types of reducing agents [such as phenolics, ascorbic acid, amino acids, organic*

acids, sugars, starch etc. etc.]. Accordingly, one gets highly heterogenous Ag-nanoparticles. If use of plants extracts has any significance, the same could have been used for commercial generation of Ag-nanoparticles. The same has been referred in our previous publications [including those appeared in PLoS One]. We could have given more specific comments, if this knowledgeable Reviewer could have pointed specific publications.

R1 Comment: "• Please check "miccrocubes""

***Response**: Our aim is simple [i.e. furnishing a rapid and simple protocol for testing pollen viability]. We don't find any relevance for testing "microcubes" [we don't believe in blindly copying, what others have reported, until and unless it has relevance].*

R1 Comment: "• References formatting must be uniform based on journal's requirement."

***Response**: This manuscript was initially submitted to PLoS Biology and the same has been transferred directly from PLos Biology website to PLoS One website. Yes, references have now been formatted in accordance with PLoS One's requirement.*

R1 Comment: "• Authors should provide page and line numbers while revising the manuscript."

***Response**: Page and line numbers have now been included.*

Reviewer #2

R2 Comment: "The manuscript has some serious issues that need to be addressed:"

***Response**: This Reviewer 2 stated that there are some serious issues that need to be addressed. <u>If the issues raised by this Reviewer can be addressed, how can the Academic Editor take a decision to reject our manuscript, without furnishing specific/valid reasons</u>. Each of the "some serious issues" raised by this Reviewer is addressed below.*

R2 Comment:1. Experiment with a non-biological control (no pollen) wherein only Silver salt and KCl in the presence of light should be conducted.

***Response**: This was very much carried out, but inclusion of this information doesn't add any significant addition to the manuscript. Further in annotated manuscript we deleted our findings with KCl.*

R2 Comment:2. Experiment with a non-biological control (no pollen) wherein only Silver salt and KCl in the absence of light should also be conducted. Please refer to the paper wherein SNPs (AgNPs) and GNPs were generated with heat-killed date palm pollens in the dark.

Response*: This was also very much carried out and we did categorically state in our manuscript that "on mixing $AgNO_3$ with KCl, an opaque white colloidal solution was produced as a result of formation of AgCl within a short duration". Therefore, we used KNO_3 and not KCl, although we do get similar results with KCl. The same has been stated in Materials and methods. Unfortunately, we have mistakenly referred KNO_3 as KCl. We apologize for the same.*

We have carefully gone through the paper of Banu et al. (2018) referred by this reviewer and noted that (i) heat-killed date palm pollen were not directly used for generation of Ag and Au nanoparticles; (ii) the extract obtained by heating stored pollen dust, was used for generating nanoparticles in dark; and (iii) investigations were carried with the presumption that the extract contains a range of phytochemicals. No attempts were made by these authors to evaluate if pollen themselves can generate nanoparticles. Hundreds of papers have been published where researchers demonstrated potential of extracts of plant parts of various plant species to generate nanoparticles. We don't see any reason, why we need to cite this paper as (i) pollen were not directly used for generation of nanoparticles; and (ii) the authors showed photos of dried inflorescence of date palm as a figure and not of pollen. Further, the findings presented in Banu et al. (2018) are nothing to do with pollen viability or any way closure to the hypothesis based on which we performed our investigations presented in our manuscript. As stated previously, we wish to again stress that our aim was not to generate Ag-nanoparticles. Unfortunately, this expert reviewer seems to be interested only in generation of Ag-nanoparticles, that too using extracts of plants parts. Our research team never believed in blindly using extracts [which contain wide range of biomolecules with different degrees of reducing capacity] for generating metal nanoparticles.

R2 Comment: "3. There are ample reports describing synthesis of SNPs using whole cell, cell free extract, plant extract for synthesis of nanoparticles using Ag salts only. I would like to know why KCl was used in the reaction as it is well known that Ag salt will be precipitated as Ag chloride."

Response*: This comment clearly reflects that this Reviewer as well as the Academic Editor Dr. Vijay Kumar have gone through our manuscript very casually, overlooking the key goal of the*

manuscript and contents elaborated in our manuscript. Our research team itself has reported potential of chloroplasts and root cell (in particular) epidermal cells generate Ag-nanoparticles. Use of plant extracts to generate AgNPs is known from last three decades. **Objective of our investigations is not to develop any AgNPs generating protocol***. We specifically stated that our objective was to test if rapid AgNPs synthesis capacity of live pollen can be exploited for testing pollen viability.*

In our original manuscript, we had categorically stated that "on mixing $AgNO_3$ with KCl, an opaque white colloidal solution was produced as a result of formation of AgCl within a short duration". This itself reflects that we are very well aware that Ag^+ precipitates as AgCl. That is the precise reason, why we stated in Materials and Methods "Therefore, it is important to disclose specifically that 1 mM $AgNO_3$ and 600 mM KCl solutions must be stored separately and former solution be added soon after pollen were incubated in later solution." Although, we were getting ideal results with KNO_3 (which we used for all our investigatiosn presented in the manuscript), we gave this information as we were getting equally good results with KCl. However, through the above referred statement, we wanted researchers (who prefer using KCl) to know that they should incubate pollen first in KCl and then add $AgNO_3$. This allows K^+ and Cl^- to be absorbed by the pollen grains before Ag^+. Moreover, the concentration of Ag^+ used was 600 times lower than that of KCl. Further, it is important to disclose true facts to the scientific community. If we wanted, we could have avoided disclosing results we obtained with KCl. To avoid any sort of confusion, now we deleted the findings with KCl in annotated manuscript.

R2 Comment: "4. Experiment using Ag salts and pollen may also be conducted."

Response*: It looks that due to casualness and lack of desired knowledge of pollen biology, this comment was made. We have specifically stated in our manuscript [based on authentic reports from several researchers, besides common observation made by even school kids studying pollen germination/biology], the significance of using an osmotic component to prevent bursting of pollen. We also stated categorically that, we diluted viability medium with pollen incubated in it, to burst the pollen so that the contents get released due to bursting. Therefore, performing any investigations of pollen viability just with silver salt is unscientific and senseless effort as live pollen burst due to severe osmotic shock (extreme hypotonic condition). However, if someone is interested in generating Ag-nanoparticles, such an effort would be ideal. It looks that both this*

reviewer and the Academic Editor, Dr. Vijay Kumar seems to be experts and only interested in generation of AgNPs and not concerned about the key objective of testing pollen viability. We feel extremely sorry to state that the Academic Editor Dr. Vijay Kumar and this Reviewer should have not taken up this assignment of evaluating our manuscript as they lacked basic background of pollen biology. Both of them seems to be probably experts in generation of AgNPs using plant extracts orelse they are extremely biased due to reasons best known to them.

R2 Comment: "5. Authors have not divulged details on various accounts, e.g. sonication conditions, heat-killing treatment and aged pollen."

Response*: As stated previously, we submitted this manuscript to PLoS Biology as a Brief Communication and hence didn't elaborate on "sonication conditions, heat-killing treatment and aged pollen". Now, we included the same in our annotated manuscript.*

R2 Comment:6. Please see the annotated pdf for detailed comments.

Suggestion of the Reviewer 2: "Subsequent to pollen rupturing" instead of "Subsequent to rupturing pollen".

Response*: We don't accept this suggestion as it gives a wrong impression. It only reflects that the Reviewer 2 seems to be not competent enough to understand the basics of pollen biology and the objective of our manuscript.*

In addition, more or less similar comments as above were marked on the PDF. The same have been extracted in JPG format and pasted below along with our specific response:

R2 Comment on PDF:

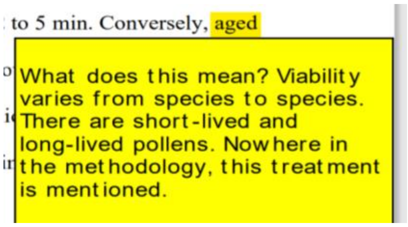
to 5 min. Conversely, aged

What does this mean? Viability varies from species to species. There are short-lived and long-lived pollens. Nowhere in the methodology, this treatment is mentioned.

Response*: It is well known/established fact that viability of pollen varies from species to species. Pollen tend to lose viability on storage, especially under ambient conditions. The term "aged" was used by us for the pollen stored under ambient conditions for different time intervals. To avoid*

confusion, now we deleted the term "aged" and stated the duration of storage in the annotated manuscript.

R2 Comment on PDF:

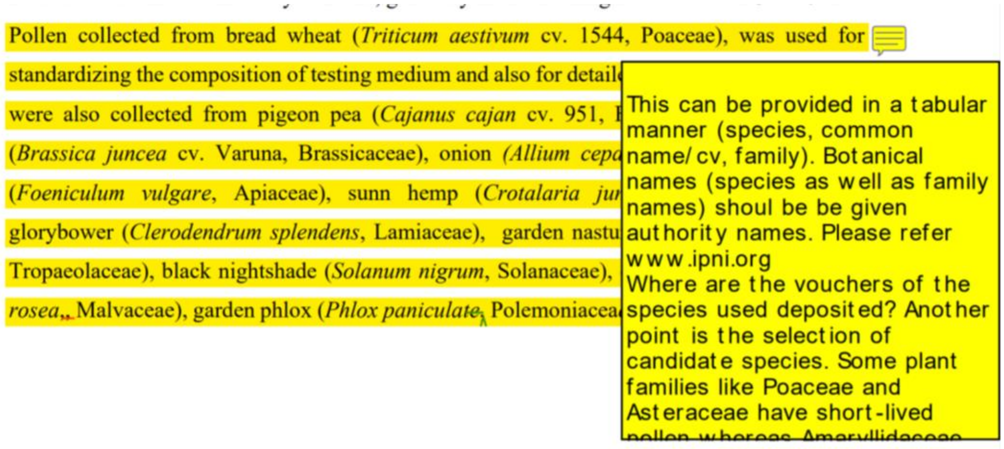

Pollen collected from bread wheat (*Triticum aestivum* cv. 1544, Poaceae), was used for standardizing the composition of testing medium and also for detail[illegible] were also collected from pigeon pea (*Cajanus cajan* cv. 951, [illegible] (*Brassica juncea* cv. Varuna, Brassicaceae), onion (*Allium cepa*[illegible] (*Foeniculum vulgare*, Apiaceae), sunn hemp (*Crotalaria ju*[illegible] glorybower (*Clerodendrum splendens*, Lamiaceae), garden nastu[illegible] Tropaeolaceae), black nightshade (*Solanum nigrum*, Solanaceae), [illegible] *rosea*, Malvaceae), garden phlox (*Phlox paniculate*, Polemoniacea[illegible]

This can be provided in a tabular manner (species, common name/ cv, family). Botanical names (species as well as family names) shoul be be given authority names. Please refer www.ipni.org
Where are the vouchers of the species used deposited? Another point is the selection of candidate species. Some plant families like Poaceae and Asteraceae have short-lived pollen whereas Amaryllidaceae [illegible]

***Response**: These comments are irrelevant and only reflects not only biased attitude as this reviewer seems to have some personal problem with us or he/she has some unknown personal gain through rejection. I don't see any valid reason why we need to unnecessarily add a Table to increase the length of the manuscript. There is absolutely nothing wrong in simply listing the plant species used. However, authority name has now been included. It is irrelevant to refer to www.ipni.org as there are superior sites.*

The question of "where are the vouchers of the species used deposited?", only reflects negative mindset of the Reviewer [as well as the Academic Editor Dr. Vijay Kumar", who entertained such incompetent comments]. Can Dr. Vijay Kumar let me know what sort of vouchers this Reviewer is referring to? Does every author who is using a plant species authorized to submit such vouchers? With regard to the reason, why we selected different plant species, we made it crystal clear in our original manuscript that "In order to check, if Ag^{+} viability medium can be used for testing pollen viability in all plant species, we extended our studies to 20 plant species ....." besides their availability. We are afraid that this Reviewer is either biased or doesn't understand simple language. It looks that both this Reviewer as well as Academic Editor believe in promoting lengthy manuscripts filled with useless packing material, rather than having crisp manuscript.

R2 Comment on PDF:

(i) on mixing $AgNO_3$ with KCl, an opaque white colloidal solution was produced as

fo [illegible] (ii) with duration of storage, efficacy of
Cl [illegible] ly brown decreased. Best, authentic and
bl [illegible] ended in 100 µl of 600 mM KCl were
h [illegible] of KCl and $AgNO_3$ is hereafter referred
bi [illegible] ctive concentration of $AgNO_3$ and KCl
a [illegible] important to disclose specifically that 1
O_3 [illegible] separately and former solution be added
po [illegible]

Formation of precipiate of AgCl is quite obvious when any silver salt is mixed with any chloride. After exposure to light, authors claim that AgCl is converted to AgNPs. However, to confirm the presence of AgNPs few more characterization techniques are needed, such as SEM, AFM to study the surface formation of AgNPs on pollens.

***Response**: That is the precise reason, why we categorically stated the same in our manuscript. We disagree that it is essential to use SEM, AFM to confirm the presence of AgNPs. I am shocked. It is not clear how AFM confirms the presence of AgNPs. To the best of our knowledge Transmission electron microscope coupled with the hardware for (a) energy dispersive X-ray (EDX) analysis; and (b) selected area electron diffraction (SAED) pattern analysis, is good enough to characterize AgNPs. In addition, we carried out vital PXRD analysis.*

R2 Comment on PDF:

Evaluating novel Ag^+ medium for testing pollen viability:

[illegible] viability medium (
[illegible] ght with a photor
[illegible] litions. In order tc
[illegible] pollen cells to br
[illegible] cubated in dark. S
[illegible] trinocular micros
[illegible] es exposed to diff
[illegible] us Imaging Ame
[illegible] o incubation, the

I am of the opinion that one more control should have been done wherein there would be no pollen (non-biological control). Please refer the article Banu, H., Renuka, N., Faheem, S. et al. Gold and Silver Nanoparticles Biomimetically Synthesized Using Date Palm Pollen Extract-Induce Apoptosis and Regulate p53 and Bcl-2 Expression in Human Breast Adenocarcinoma Cells. Biol Trace Elem Res 186, [illegible]

***Response**: This statement of the Reviewer, clearly reflects the way he/she can mislead the scientific world. We have carefully gone through the paper of Banu et al. (2018) referred by this reviewer and noted that (i) heat-killed date palm pollen were not directly used for generation of Ag and Au nanoparticles; (ii) the extract obtained by heating stored pollen dust, was used for generating nanoparticles in dark; and (iii) investigations were carried with the presumption that the extract contains a range of phytochemicals. No attempts were made by these authors to evaluate if pollen*

themselves can generate nanoparticles. The authors showed photos of dried inflorescence of date palm as a figure and not of pollen. Further, the findings presented in Banu et al. (2018) are nothing to do with pollen viability or any way closure to the hypothesis based on which we performed our investigations presented in our manuscript. As stated previously, we wish to again stress that our aim was not to generate Ag-nanoparticles. Unfortunately, this expert reviewer seems to be interested only in generation of Ag-nanoparticles, that too using extracts of plants parts. Our research team never believed in blindly using extracts [which contain wide range of biomolecules with different degrees of reducing capacity] for generating metal nanoparticles.

R2 Comment on PDF:

Original: “grasped”

Reviewer 2’s suggestion: “captured”

Response*: This suggestion has been incorporated in the annotated manuscript..*

R2 Comment on PDF:

sonication conditions may be mentioned

Response*: Sonication conditions have been included in the annotated manuscript.*

R2 Comment on PDF:

Previously, we reported potential of (i) live leaf cells; and (ii) chloroplasts to reduce Ag^+

Few control experiments need to be done like the silver salt +chloride without pollens (nonbiological control) and one with pollens + silver salt without chlorides and characterization of outcomes of these experiments if these solutions still turn brown.

ation or high intensity light (~600 µmol m^{-2} s^{-1} PF[illegible]

illed wheat pollen incubated in Ag^+ viability mediu[illegible]

ensity light failed to turn brown (~~fig.~~ 1D), reveali[illegible]

ollen for rapid generation of AgNPs.

m can be used for testing pollen viability in all pla[illegible]

plant species (~~namely~~ pigeon pea, Indian musta[illegible]

r, garden nasturtium, black nightshade, comm[illegible]

Response: These statements again reflects that this Reviewer has not gone through our manuscript carefully. He could not grasp basic objective, hypothesis and the novel findings of the investigations presented in our manuscript.

R2 Comment on PDF:

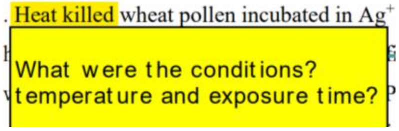
. Heat killed wheat pollen incubated in Ag^+

What were the conditions?
temperature and exposure time?

Response*: Details of how pollen was heat killed has now been elaborated in the annotated manuscript.*

R2 Comment on PDF: Names of all Journals have been highlighted as they were represented in full form rather than in abbreviated form.

Response*: Finally, Reviewer 2 given his comment against name of every journal in Reference section, as full name of every journal was given instead of in abbreviated form. As stated previously, our manuscript was transferred directly from PLoS Biology website to PLoS One website as per the accepted terms and conditions. Therefore, the format of references was in PLoS Biology style. Names of Journals can easily be abbreviated. This can't be taken as a criterion for rejection of any manuscript.*

We have now abbreviated the names of the journals in the annotated manuscript.

.
